# Predicting poor response to anti-osteoporosis therapy: a machine learning model integrating clinical and novel biomarker data

**DOI:** 10.3389/fmed.2026.1786209

**Published:** 2026-05-14

**Authors:** Yannan Bi, Maolin Zhang, Weiqiong Zhang, Jiahong Li

**Affiliations:** Department of Orthopedics, The Fourth Affiliated Hospital, Guangzhou Medical University, Guangzhou, Guangdong, China

**Keywords:** biomarkers, machine learning, osteoporosis, prediction model, treatment response prediction

## Abstract

**Objective:**

This study was conducted to develop and validate a prediction model integrating clinical characteristics and novel biomarkers. The goal was to identify patients at high risk for a poor response to standard anti-osteoporosis therapy prior to treatment initiation, thereby supporting personalized therapeutic decision-making.

**Methods:**

A retrospective analysis was performed on 543 patients with primary osteoporosis admitted between January 2021 and December 2024. All patients received 12 months of standard treatment. Participants were randomly allocated to a training set (*n* = 380) and a validation set (*n* = 163) in a 7:3 ratio. In the training set, univariate analysis, Least Absolute Shrinkage and Selection Operator (LASSO) regression, and multivariate logistic regression were used to determine independent predictors. Three machine learning models—Random Forest (RF), Support Vector Machine (SVM), and K-Nearest Neighbors (KNN)—were then constructed. Model performance was evaluated using the area under the receiver operating characteristic curve (AUC), calibration curves, and decision curve analysis (DCA). SHapley Additive exPlanations (SHAP) values were used to interpret the optimal model.

**Results:**

Baseline characteristics were comparable between the training and validation sets (*P* > 0.05). Eight independent predictors of poor treatment response were identified: comorbid diabetes, history of fragility fracture, glucocorticoid use for ≥ 6 months, femoral neck bone mineral density T-score, and serum levels of osteocalcin, procollagen type I N-terminal propeptide, β-CrossLaps of type I collagen (β-CTX), and 25-Hydroxyvitamin D. Among the models, the RF algorithm demonstrated superior performance, with an AUC of 0.856 (95% CI: 0.808–0.905) in the training set and 0.825 (95% CI: 0.718–0.933) in the validation set. The model was well-calibrated, and DCA indicated a high net benefit. SHAP analysis confirmed serum β-CTX as the most significant predictive variable.

**Conclusion:**

A predictive model integrating multi-dimensional factors was successfully developed and validated for assessing osteoporosis treatment efficacy. The RF-based model exhibited robust predictive performance and clinical utility. It shows potential for pre-therapeutic identification of high-risk patients, facilitating precision management in osteoporosis.

## Introduction

Osteoporosis is a systemic skeletal disease characterized by low bone mass and microarchitectural deterioration of bone tissue, leading to increased bone fragility and susceptibility to fracture (1). With the intensifying global aging population, osteoporosis and its associated fragility fractures have become major public health issues, imposing a heavy burden on patients, families, and the healthcare system (2). Currently, anti-osteoporotic medications such as bisphosphonates and parathyroid hormone analogs are the primary treatments, aiming to increase bone mineral density (BMD) and reduce fracture risk. However, patient responses to treatment exhibit significant heterogeneity in clinical practice (3). Studies indicate that even with standard treatment, a considerable proportion of patients show suboptimal BMD improvement or experience new fractures, a condition termed “inadequate treatment response”. This variability in efficacy may be related to patients’ complex clinical characteristics, bone metabolic status, and drug responsiveness (4). Presently, clinicians primarily rely on baseline BMD and limited conventional bone turnover markers, such as β-CrossLaps of Type I Collagen (β-CTX) and Procollagen Type I N-terminal Propeptide (PINP), to assess disease status and treatment response. A predictive tool for comprehensively evaluating individual efficacy risk before treatment initiation is lacking.

In recent years, a series of novel biomarkers involved in bone metabolism regulation, such as Dickkopf-1 (DKK1), osteoprotegerin (OPG), and Receptor Activator of Nuclear Factor-κB Ligand (RANKL), have garnered attention. These biomarkers reflect the balance between bone resorption and formation through different pathways (5). However, the predictive value of a single biomarker is limited. No study has systematically integrated these novel biomarkers with patient clinical characteristics to construct a comprehensive prediction model. Machine learning methods, capable of handling high-dimensional data and capturing complex non-linear relationships among variables, have been widely applied in developing medical prediction models.

This study aims to conduct a retrospective cohort study to systematically collect clinical data and a series of novel biomarker data from osteoporosis patients. Core predictive variables will first be screened using univariate analysis and Least Absolute Shrinkage and Selection Operator (LASSO) regression. Subsequently, multivariate logistic regression will be employed to identify independent influencing factors. Based on these factors, various machine learning prediction models will be constructed. Through rigorous internal validation and performance evaluation—including discrimination, calibration, and clinical utility—and utilizing SHAP values to enhance model interpretability, this study seeks to develop a practical tool for accurately predicting individualized efficacy risk before treatment initiation. This tool aims to provide a basis for achieving precision medicine in osteoporosis management.

## Materials and methods

### Study population

This single-center retrospective cohort study consecutively enrolled patients diagnosed with primary osteoporosis who initiated standard anti-osteoporosis treatment at osteoporosis specialty clinic or orthopedics inpatient department of the Third Affiliated Hospital, Guangzhou Medical University, and the Fifth Affiliated Hospital, Guangzhou Medical University between January 2021 and December 2024. The study protocol was approved by the Hospital Ethics Review Board, and all patients provided written informed consent.

Inclusion criteria were: (1) Age 50–85 years. (2) Meeting the diagnostic criteria for primary osteoporosis as stated above (6). (3) First-time initiation of anti-osteoporosis drug therapy. (4) Planned to receive continuous standard treatment for ≥ 12 months. (5) Completion of dual-energy X-ray absorptiometry (DXA) BMD and specified biomarker tests both before treatment and 12 months after treatment. (6) Complete clinical and follow-up data.

Exclusion criteria were: (1) Secondary osteoporosis (e.g., hyperparathyroidism, renal insufficiency, malignant bone metastases). (2) Use of other medications affecting bone metabolism within the past 6 months (e.g., sex hormones, fluoride, diuretics; except for documented glucocorticoid use recorded as a study variable). (3) Comorbid severe cardiac, hepatic, or renal failure. (4) Advanced malignant tumor or life expectancy less than 1 year. (5) Presence of psychiatric illness or cognitive impairment preventing cooperation. (6) Missing baseline BMD or biomarker data.

Based on preliminary data, the anticipated incidence of the primary outcome (inadequate treatment response) was set at 20–25%. Sample size estimation was performed using PASS 2021 software and the R language “pwr” package. With a significance level (α) of 0.05, statistical power (1-β) of 80%, and consideration of a 10% data missing rate, the minimum required sample size was calculated to be 486.

All enrolled patients were randomly divided in a 7:3 ratio into a training set (*n* = 380) and a validation set (*n* = 163) using a complete randomization method.

### Treatment protocol

All patients selected and received one of the following standard anti-osteoporosis drug regimens based on their condition: (1) Bisphosphonates (alendronate 70 mg/week orally, or zoledronic acid 5 mg/year intravenously); (2) Calcitonin (salmon calcitonin 50 IU/injection, intramuscularly once daily for 2 weeks, then changed to every other day); (3) Parathyroid hormone analogs (teriparatide 20 μg/day subcutaneously). All patients concurrently received calcium supplements (elemental calcium 800–1,000 mg/day) and vitamin D (400–800 IU/day). The total treatment course was 12 months.

### Data collection

Data were collected from the hospital’s electronic medical record system, laboratory information system, and specialized disease database.

Baseline Clinical Data: Included age, sex, height, weight (for calculating Body Mass Index, BMI), smoking history (defined as smoking ≥ 10 cigarettes/day for ≥ 5 years), alcohol consumption history (defined as consuming alcohol ≥ 50 g/day for ≥ 5 years), comorbidities (hypertension, type 2 diabetes, coronary heart disease), history of prior fragility fracture, and history of continuous glucocorticoid use (≥ 6 months).

Bone Mineral Density Measurement: Before treatment, BMD at the lumbar spine (L1-L4), femoral neck, and total hip was measured using a dual-energy X-ray absorptiometer (Hologic Discovery Wi, United States), and T-scores were recorded. The instrument was calibrated before measurements to ensure a coefficient of variation < 1%.

Biomarker Measurement: Before treatment, fasting venous blood was collected from patients in the morning. Serum was separated by centrifugation and stored at -80°C until assayed. Serum osteocalcin (OC), PINP, and β-CTX were measured using electrochemiluminescence (Roche Cobas e601 analyzer). Serum 25-Hydroxyvitamin D (25(OH)D) was measured using high-performance liquid chromatography (Shimadzu LC-20AT system). Serum DKK1, sclerostin, OPG, and RANKL were measured using enzyme-linked immunosorbent assay. All procedures strictly followed the reagent instructions. Each sample was tested in triplicate, and the average value was used.

To reduce batch effects and inter-assay variability in the detection process, strict laboratory quality control measures were adopted in this study: (1) All serum samples were divided into detection batches according to the same standard, and the same batch of quality control products (with known concentration values) were added to each detection batch for parallel detection; (2) The coefficient of variation (CV) of the quality control products was controlled within 10%, and the detection data of the batch were discarded and re-detected if the CV exceeded the threshold; (3) The same operator completed the detection of the same type of biomarker, and the detection instruments (Roche Cobas e601, Shimadzu LC-20AT) were calibrated and maintained regularly before each batch of detection; (4) All samples were tested in triplicate, and the average value was taken as the final detection result to reduce the random error of a single detection.

### Outcome definition and grouping

The primary outcome was treatment efficacy after 12 months of standard anti-osteoporosis therapy, which was defined by integrating biological response (BMD change) and clinical outcomes (fracture and treatment adjustment) to comprehensively reflect the actual therapeutic effect in clinical practice. Patients were divided into two groups:

Effective treatment group: According to the recommendations of the American Association of Clinical Endocrinologists/American College of Endocrinology Clinical Practice Guidelines for the Diagnosis and Treatment of Postmenopausal Osteoporosis (2020 Update), patients who, after 12 months of treatment, showed a BMD T-score increase of ≥ 3% at the primary measurement site (lumbar spine or hip) compared to baseline, and had no new fragility fractures were defined as the effective treatment group.

Ineffective treatment group: Patients meeting any of the following criteria: (1) After 12 months of treatment, the BMD T-score increase at the primary measurement site was < 3% compared to baseline; (2) Occurrence of a new fragility fracture during the treatment period (confirmed by imaging); (3) Change of treatment regimen within 12 months due to poor efficacy or intolerable adverse reactions.

Efficacy assessment was independently performed by two researchers blinded to the patients’ baseline biomarker levels. In case of disagreement, a third senior expert made the final decision.

### Statistical analysis

Data analysis was performed using SPSS 26.0, R 4.2.3, and Python 3.8.5 software. Normally distributed continuous data are presented as mean ± standard deviation and compared between groups using the independent samples *t*-test. Non-normally distributed data are presented as median (interquartile range) and compared using the Mann-Whitney U test. Categorical data are presented as number (percentage) and compared using the χ^2^ test or Fisher’s exact test.

In the training set, univariate analysis was first conducted to screen variables associated with treatment efficacy. These statistically significant variables were then incorporated into a LASSO regression model for variable compression and feature selection. The optimal penalty coefficient (λ) was determined using 10-fold cross-validation. The variables selected by LASSO were further included in a multivariate logistic regression analysis to calculate the odds ratio (OR) and 95% confidence interval (CI) for each variable, thereby identifying independent influencing factors for inadequate treatment response.

Based on the final variable set identified by the multivariate logistic regression, three machine learning prediction models were constructed: Random Forest (RF), Support Vector Machine (SVM) (with radial basis function kernel), and K-Nearest Neighbors (KNN). Model hyperparameters were strictly optimized within the training set to avoid overfitting, and the specific tuning process was as follows: for the Random Forest model, the number of decision trees (n_estimators) and the maximum depth of the tree (max_depth) were the main optimized hyperparameters, with the search range of n_estimators set as 100–500 and max_depth set as 5–20; for the SVM model with radial basis function kernel, the penalty coefficient (C) and kernel function parameter (gamma) were optimized, with the search range of C set as 0.1–10 and gamma set as 0.001–1; for the KNN model, the number of nearest neighbors (n_neighbors) was optimized, with the search range set as 3–15. All hyperparameters were optimized by 10-fold cross-validation within the training set, and the combination of hyperparameters with the highest area under the curve (AUC) value in the cross-validation was selected as the optimal hyperparameter setting for each model (Supplementary material).

Model performance was evaluated in both the training and validation sets: The AUC of receiver operating characteristic (ROC) was used to assess discrimination. Calibration curves were plotted, and the Brier score was calculated; the Hosmer-Lemeshow test was performed to assess calibration. Decision curve analysis (DCA) plots were drawn to evaluate clinical net benefit. For the best-performing model, SHapley Additive exPlanations (SHAP) values were used for interpretability analysis. All statistical tests were two-sided, and a *P* < 0.05 was considered statistically significant.

## Results

### Comparison of baseline characteristics between training and validation sets

No statistically significant differences (*P* > 0.05) were observed regarding gender, age, BMI, smoking history, alcohol consumption history, comorbidities, history of prior fragility fracture, glucocorticoid use history, BMD, and biomarker levels between the training and validation sets, indicating comparability (Table 1).

**TABLE 1:** Comparison of baseline characteristics between the training and validation sets.

Variables	Training set (*n* = 380)	Validation set (*n* = 163)	*t*/χ *^2^*	*P*
Age (years)	67.36 ± 8.13	67.85 ± 7.96	0.647	0.517
BMI (kg/m^2^)	22.35 ± 3.46	22.46 ± 3.51	0.338	0.735
Gender (male/female)	105(27.63%)/275(72.37%)	44(27.00%)/119(73.00%)	0.023	0.878
Smoking history (yes/no)	78(20.53%)/302(79.47%)	33(20.25%)/130(79.75%)	0.005	0.940
Alcohol use history (yes/no)	46(12.11%)/334(87.89%)	20(12.27%)/143(87.73%)	0.002	0.957
Comorbid hypertension (yes/no)	154(40.53%)/227(59.74%)	67(41.10%)/96(58.90%)	0.022	0.881
Comorbid diabetes (yes/no)	89 (23.42%)/291(76.58%)	37(22.70%)/126(77.30%)	0.033	0.855
Comorbid coronary heart disease (yes/no)	70(18.42%)/310(81.58%)	32(19.63%)/131(80.37%)	0.109	0.740
History of fragility fracture (yes/no)	107(28.16%)/273(71.84%)	43(26.38%)/120(73.62%)	0.180	0.671
Glucocorticoid use ≥ 6 months (yes/no)	80(21.05%)/300(78.95%)	32(19.63%)/131(80.37%)	0.141	0.708
Lumbar spine BMD T-score	−3.01 ± 0.72	−2.94 ± 0.82	0.995	0.320
Femoral neck BMD T-score	−2.73 ± 0.60	−2.68 ± 0.71	0.841	0.400
Total hip BMD T-score	−2.51 ± 0.53	−2.48 ± 0.61	0.577	0.564
Serum OC (ng/mL)	18.14 ± 5.25	17.82 ± 5.04	0.658	0.510
Serum PINP (ng/mL)	35.52 ± 10.15	34.85 ± 9.93	0.709	0.478
Serum β-CTX (ng/mL)	0.44 ± 0.14	0.43 ± 0.15	0.747	0.456
Serum 25(OH)D (ng/mL)	22.42 ± 6.74	22.05 ± 6.52	0.582	0.554
Serum DKK1 (pg/mL)	377.56 ± 88.63	372.92 ± 91.47	0.554	0.580
Serum sclerostin (ng/mL)	0.87 ± 0.24	0.86 ± 0.26	0.434	0.665
Serum OPG (pg/mL)	425.67 ± 95.34	421.82 ± 93.75	0.433	0.664
Serum RANKL (pg/mL)	315.45 ± 78.56	312.62 ± 76.98	0.387	0.698
Treatment regimen			0.007	0.996
Bisphosphonates	266(70.00%)	114(69.94%)		
Calcitonin analogs	76(20.00%)	33(20.24%)		
Parathyroid hormone analogs	38(10.00%)	16(9.82%)		

### Univariate analysis of osteoporosis treatment efficacy

In the training set of 380 patients, 79 cases (20.79%) exhibited poor treatment response, while 301 cases (79.21%) were treatment-responsive. In the 79 cases of poor treatment response in the training set, the specific composition of the outcome events was as follows: 58 cases (73.42%) were due to BMD T-score increase < 3% at the primary measurement site without new fractures, 11 cases (13.92%) were due to the occurrence of new fragility fractures during treatment, and 10 cases (12.66%) involved treatment adjustment due to poor efficacy (*n* = 7, 70.00%) or intolerable adverse reactions (*n* = 3, 30.00%). Univariate analysis revealed that the presence of diabetes mellitus, history of fragility fracture, glucocorticoid use for ≥ 6 months, femoral neck BMD T-score, serum PINP, serum β-CTX, serum 25(OH)D, and serum OC were associated with poor treatment response (*P* < 0.05) (Table 2).

**TABLE 2:** Univariate analysis of treatment efficacy in the training set.

Variables	Ineffective treatment group (*n* = 79)	Effective treatment group (*n* = 301)	*t*/χ *^2^*	*P*
Age (years)	68.65 ± 7.62	66.92 ± 8.02	1.723	0.085
BMI (kg/m^2^)	21.86 ± 3.25	22.51 ± 3.52	1.483	0.138
Gender (male/female)	28(35.44%)/51(64.56%)	77(25.58%)/224(74.42%)	3.043	0.081
Smoking history (Yes/No)	16(20.25%)/63(79.75%)	62(20.60%)/239(79.40%)	0.004	0.945
Alcohol use history (yes/no)	10(12.66%)/69(87.34%)	36(11.96%)/265(88.04%)	0.028	0.865
Comorbid hypertension (yes/no)	33(41.77%)/46(58.23%)	121(40.20%)/180(59.80%)	0.064	0.800
Comorbid diabetes (yes/no)	29(36.71%)/50(63.29%)	60(19.93%)/241(80.07%)	9.818	0.001
Comorbid coronary heart disease (yes/no)	16(20.25%)/63(79.75%)	54(17.94%)/247(82.06%)	0.222	0.636
History of fragility fracture (yes/no)	33(41.77%)/46(58.23%)	74(24.58%)/227(75.42%)	9.138	0.002
Glucocorticoid Use ≥ 6 months (Yes/No)	25(31.65%)/54(68.35%)	55(18.27%)/246(81.73%)	6.733	0.009
Lumbar spine BMD T-score	−3.05 ± 0.76	−2.98 ± 0.70	0.776	0.437
Femoral neck BMD T-score	−2.98 ± 0.58	−2.65 ± 0.56	6.029	0.001
Total hip BMD T-score	−2.58 ± 0.51	−2.48 ± 0.54	1.481	0.139
Serum OC (ng/mL)	16.96 ± 4.87	18.75 ± 5.32	2.707	0.007
Serum PINP (ng/mL)	28.63 ± 9.52	37.85 ± 10.06	7.329	0.001
Serum β-CTX (ng/mL)	0.52 ± 0.17	0.42 ± 0.12	5.998	0.001
Serum 25(OH)D (ng/mL)	18.25 ± 6.23	24.17 ± 6.58	7.194	0.001
Serum DKK1 (pg/mL)	386.52 ± 92.37	372.68 ± 85.41	1.260	0.208
Serum sclerostin (ng/mL)	0.91 ± 0.27	0.86 ± 0.23	1.656	0.098
Serum OPG (pg/mL)	438.65 ± 98.24	419.72 ± 93.56	1.583	0.114
Serum RANKL (pg/mL)	328.56 ± 81.42	310.25 ± 76.38	1.870	0.062
Treatment regimen			0.006	0.996
Bisphosphonates	55(69.62%)	211(70.10%)		
Calcitonin analogs	16(20.25%)	60(19.93%)		
Parathyroid hormone analogs	8(10.13%)	30(9.97%)		

### Multivariate logistic regression analysis of factors influencing osteoporosis treatment efficacy

**FIGURE 1:**
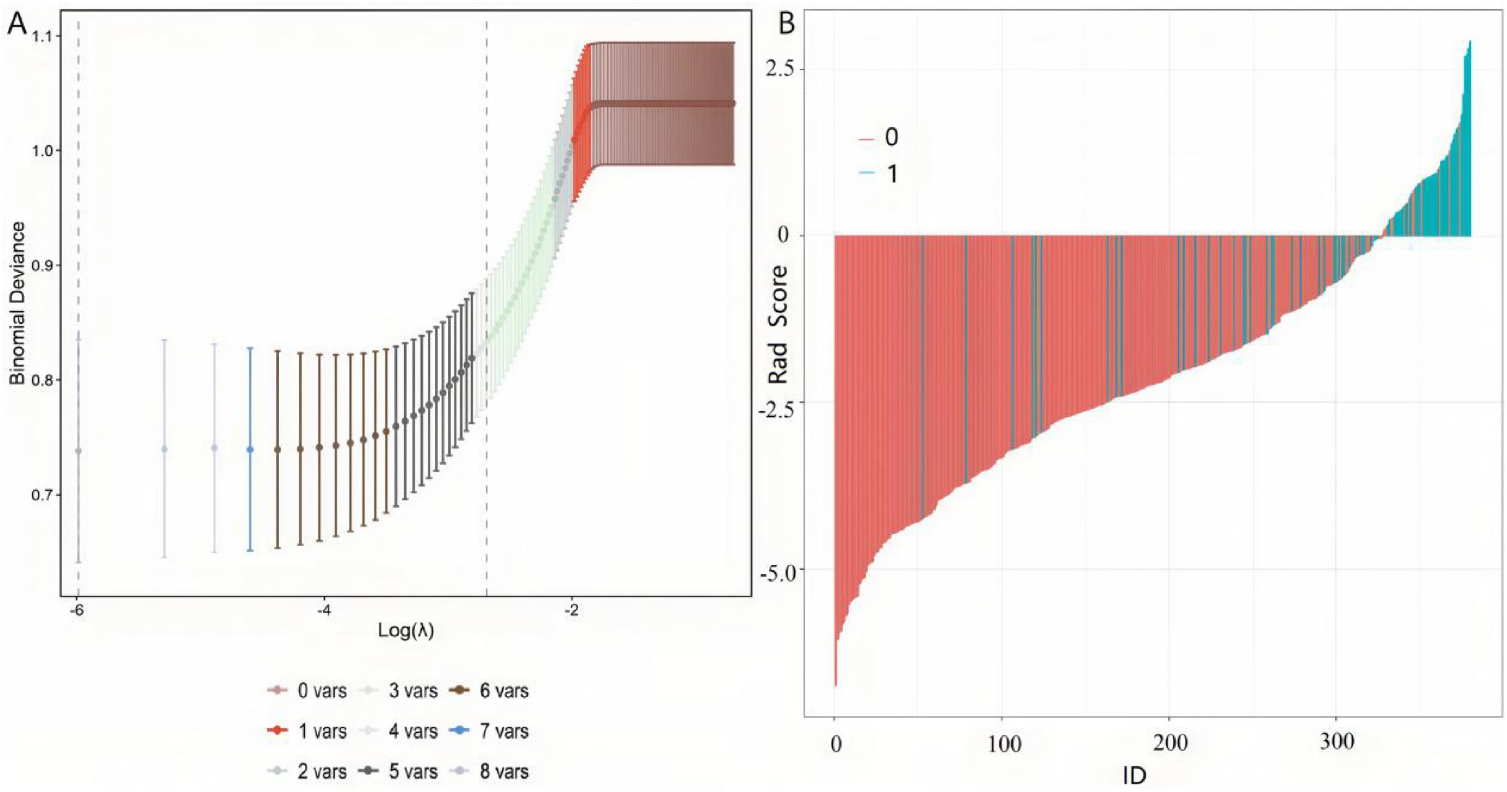
LASSO regression for variable selection. **(A)** Tuning parameter (λ) selection with 10-fold cross-validation; vertical dashed line indicates optimal λ (minimum error). **(B)** Coefficient profiles of 8 candidate variables; none were shrunk to zero at optimal λ.

The eight significant variables identified in the univariate analysis were included in a LASSO regression model for variable compression and feature selection. The optimal penalty coefficient λ (lambda.min) was determined via 10-fold cross-validation. The LASSO regression path plot demonstrated gradual shrinkage of variable coefficients as the logarithm of the penalty coefficient increased. At the optimal λ value, none of the coefficients for the eight variables were compressed to zero, indicating that all variables possessed predictive value (Figure 1).

These eight variables selected by LASSO regression were subsequently incorporated into a multivariate logistic regression analysis (Table 3). The results identified the presence of diabetes mellitus, history of fragility fracture, glucocorticoid use for ≥ 6 months, femoral neck BMD T-score, serum OC, serum PINP, serum β-CTX, and serum 25(OH)D as independent influencing factors for poor treatment response (*P* < 0.05) (Table 4).

**TABLE 3:** Variable assignment.

Variable	Meaning	Assignment
X1	Comorbid diabetes	1 = Yes, 0 = No
X2	History of fragility fracture	1 = Yes, 0 = No
X3	Glucocorticoid use ≥ 6 months	1 = Yes, 0 = No
X4	Femoral neck BMD T-score	Continuous
X5	Serum OC	Continuous
X6	Serum PINP	Continuous
X7	Serum β-CTX	Continuous
X8	Serum 25(OH)D	Continuous
Y	Osteoporosis treatment efficacy	1 = Ineffective treatment, 0 = Effective treatment

**TABLE 4:** Multivariate logistic regression analysis of factors influencing osteoporosis treatment efficacy.

Factor	β	*SE*	Wald	*P*	*OR*	95%CI
Comorbid diabetes	2.281	1.074	4.508	0.034	3.784	1.192–8.331
History of fragility fracture	0.204	0.076	7.201	0.007	1.226	1.013–1.794
Glucocorticoid use ≥ 6 months	1.442	0.885	2.652	0.013	4.228	1.746–9.974
Femoral neck BMD T-score	−0.397	0.214	3.939	0.037	0.673	0.455–0.995
Serum OC	−0.063	0.030	4.292	0.028	0.939	0.885–0.997
Serum PINP	−0.098	0.017	33.155	0.001	0.906	0.877–0.937
Serum β-CTX	4.405	1.134	15.096	0.001	5.879	3.870–10.886
Serum 25(OH)D	−0.150	0.027	31.639	0.001	0.861	0.817–0.907

### Development and evaluation of the osteoporosis treatment efficacy prediction model

**FIGURE 2:**
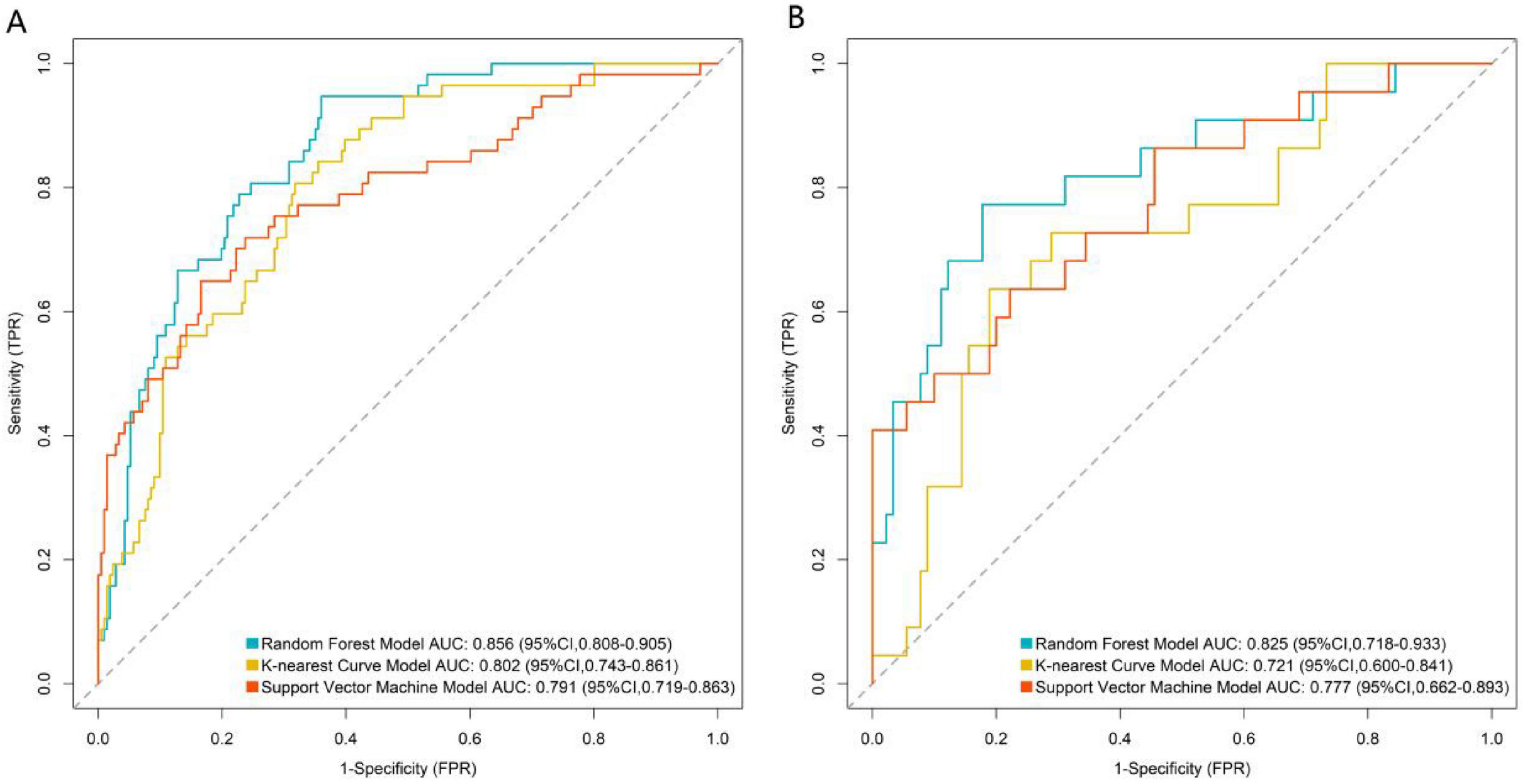
Receiver operating characteristic curve analysis of three machine learning models in the training **(A)** and validation **(B)** sets. FPR, False positive rate; TPR, True positive rate).

ROC curve analysis results (Figure 2) showed that in the training set (Figure 2A), the RF model demonstrated the strongest discriminatory ability, with an AUC of 0.856 (95% CI: 0.808–0.905). The AUCs for the KNN and SVM models were 0.802 (95% CI: 0.743–0.861) and 0.791 (95% CI: 0.719–0.863), respectively. In the independent validation set (Figure 2B), the RF model maintained the best generalization performance, with an AUC of 0.825 (95% CI: 0.718–0.933), which was higher than that of the SVM model (AUC = 0.777, 95% CI: 0.662–0.893) and the KNN model (AUC = 0.721, 95% CI: 0.600–0.841).

**FIGURE 3:**
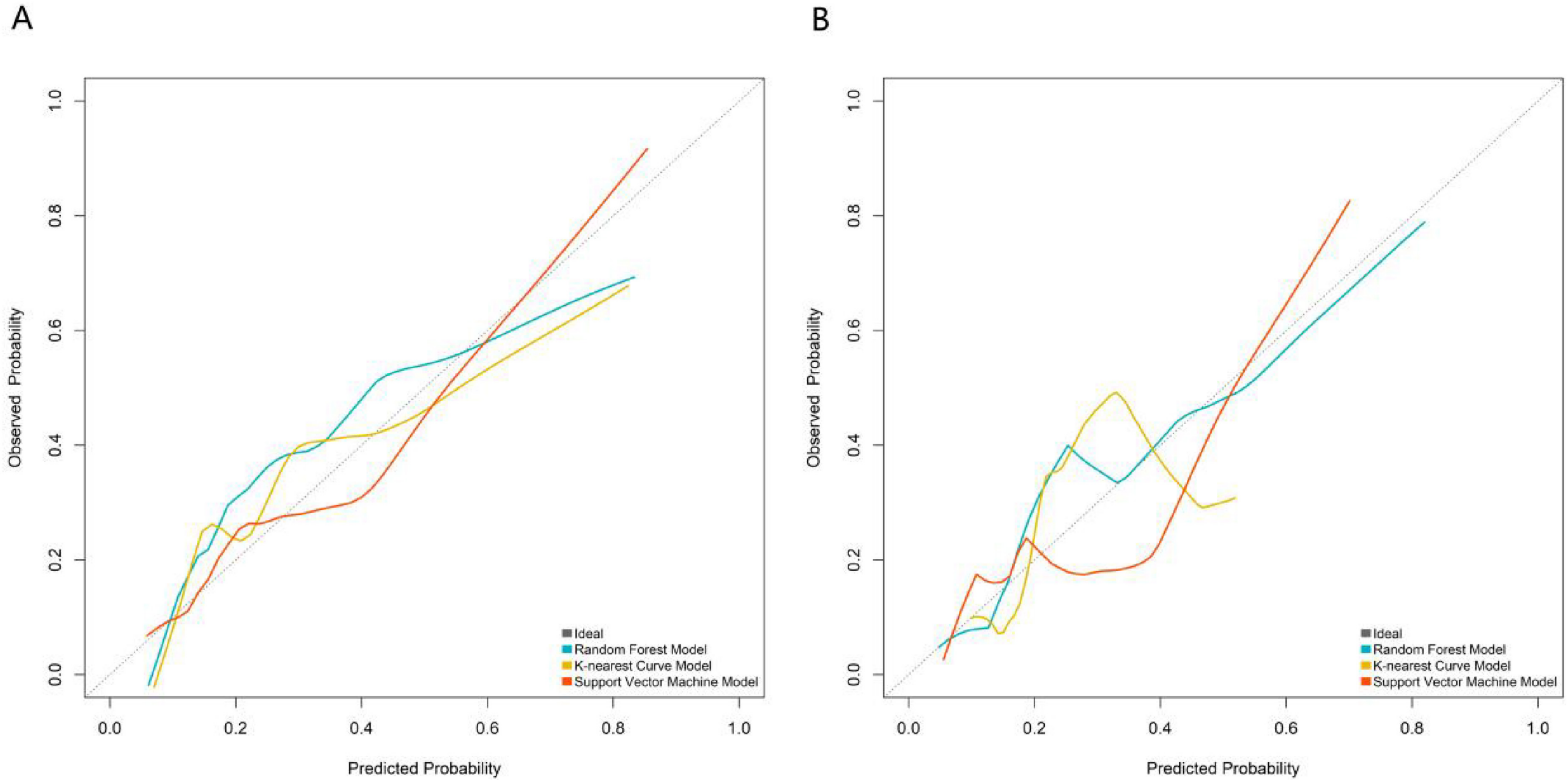
Calibration curves of the RF model of three machine learning models. **(A)** Training set: the calibration curve of the Random Forest model was closest to the ideal diagonal: Brier score = 0.132, Hosmer–Lemeshow *P* = 0.423. **(B)** Validation set: the calibration curve of the Random Forest model was closest to the ideal diagonal: Brier score = 0.148, Hosmer–Lemeshow *P* = 0.385. The curves closely follow the ideal diagonal, indicating good calibration.

In both the training set (Figure 3A) and validation set (Figure 3B), the calibration curve of the RF model was closest to the ideal diagonal. Its Brier score was 0.132 in the training set and 0.148 in the validation set. The Hosmer-Lemeshow test indicated no statistically significant difference for the RF model in either the training set (*P* = 0.423) or the validation set (*P* = 0.385), suggesting excellent agreement between predicted probabilities and actual risk, and thus optimal calibration.

**FIGURE 4:**
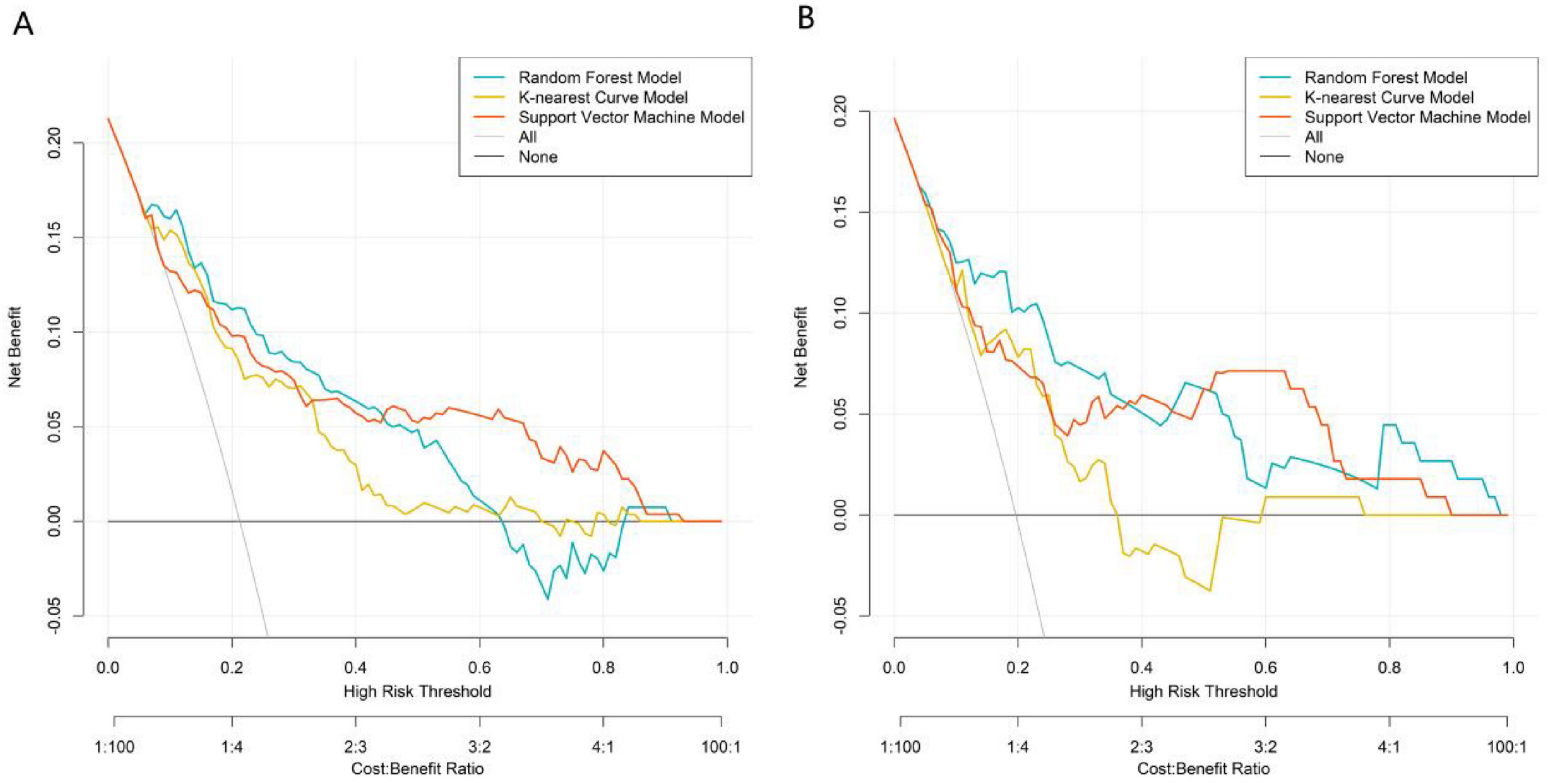
Decision curve analysis of three models. **(A)** Training set: Random Forest provides highest net benefit across threshold probabilities 0.1–0.7. **(B)** Validation set: Random Forest remains superior, supporting its clinical utility.

DCA was employed to evaluate the clinical net benefit of the models. Across a wide range of risk thresholds (approximately 0.1–0.7), the net benefit obtained by applying the three machine learning models to guide clinical decisions (i.e., intervening on patients predicted to be high-risk by the model) was superior to the extreme strategies of “treat all” and “treat none.” Within the commonly used clinical decision threshold interval (0.2–0.5), the net benefit curve of the RF model was the highest in both the training set (Figure 4A) and validation set (Figure 4B), indicating its superior clinical utility. The risk threshold range of 0.1–0.7 selected in the DCA is consistent with the clinical risk stratification standard of osteoporosis treatment in clinical practice, among which the 0.2–0.5 threshold interval is the core range for clinicians to make anti-osteoporosis treatment adjustment decisions. When the predicted risk of poor treatment response is lower than 0.2, it indicates that the patient is at low risk, and the standard treatment regimen can be continued without adjustment; when the predicted risk is between 0.2 and 0.5, it indicates that the patient is at moderate risk, and clinicians need to strengthen follow-up and adjust the treatment plan in a timely manner; when the predicted risk is higher than 0.5, it indicates that the patient is at high risk, and it is necessary to replace the more potent anti-osteoporosis drugs or adopt combined therapy to improve the treatment efficacy.

### Interpretability assessment of the model predictions

**FIGURE 5:**
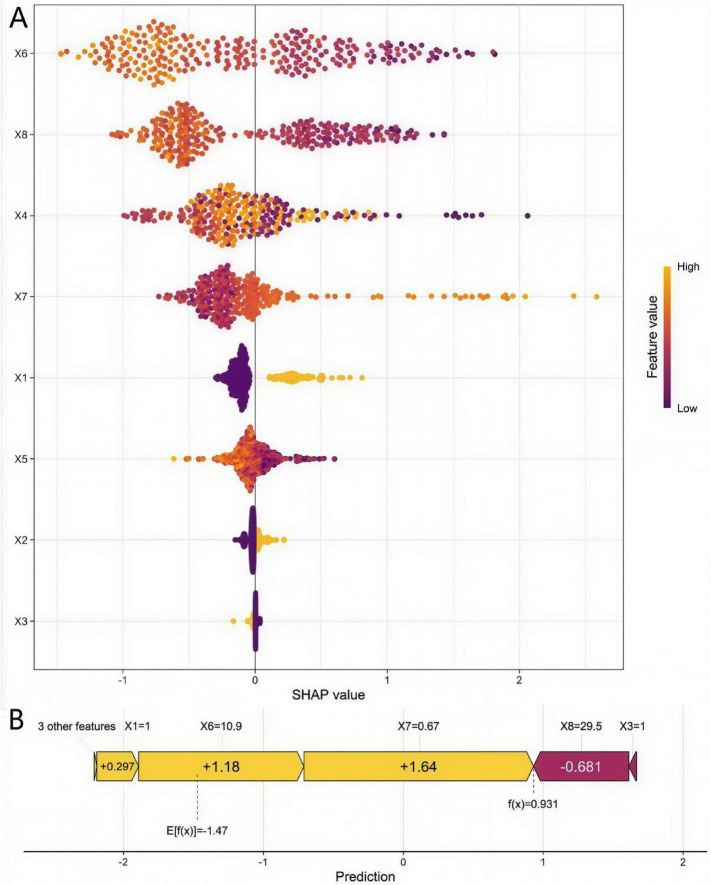
SHAP summary plot **(A)** and dependence plots **(B)**. X1, Comorbid Diabetes; X2, History of Fragility Fracture; X3, Glucocorticoid Use ≥ 6 months; X4, Femoral Neck BMD T-score; X5, Serum OC (ng/mL); X6, Serum PINP (ng/mL); X7, Serum β-CTX (ng/mL); X8, Serum 25(OH)D (ng/mL).

To enhance model transparency and clinical acceptability, SHAP values were utilized to interpret the best-performing RF model. Global feature importance analysis (Figure 5A) quantified the average contribution intensity of each variable to the prediction outcome. The ranking based on mean absolute SHAP values identified serum β-CTX (0.42) as the most important predictor, followed sequentially by serum PINP (0.38), serum 25-OH-VD (0.31), diabetes mellitus (0.22), femoral neck BMD T-score (0.18), serum OC (0.15), history of fragility fracture (0.12), and glucocorticoid use for ≥ 6 months (0.09). This ranking was highly consistent with the impact degree of variables in the multivariate logistic regression analysis.

SHAP dependence plots (Figure 5B) further elucidated the non-linear relationships between key variables and the model predictions. Taking the most important variable, serum β-CTX, as an example: when its level was below 0.35 ng/mL, the corresponding SHAP values were mostly negative, indicating that lower β-CTX levels tended to decrease the model’s predicted risk of “poor response.” When its level exceeded approximately 0.45 ng/mL, the SHAP values rapidly shifted to positive and increased substantially, suggesting that high β-CTX levels may significantly increase the predicted risk of poor treatment response in this study population. This visualization preliminarily demonstrates the potential threshold effect of this biomarker on treatment efficacy, which needs further verification by multi-center studies.

**FIGURE 6:**
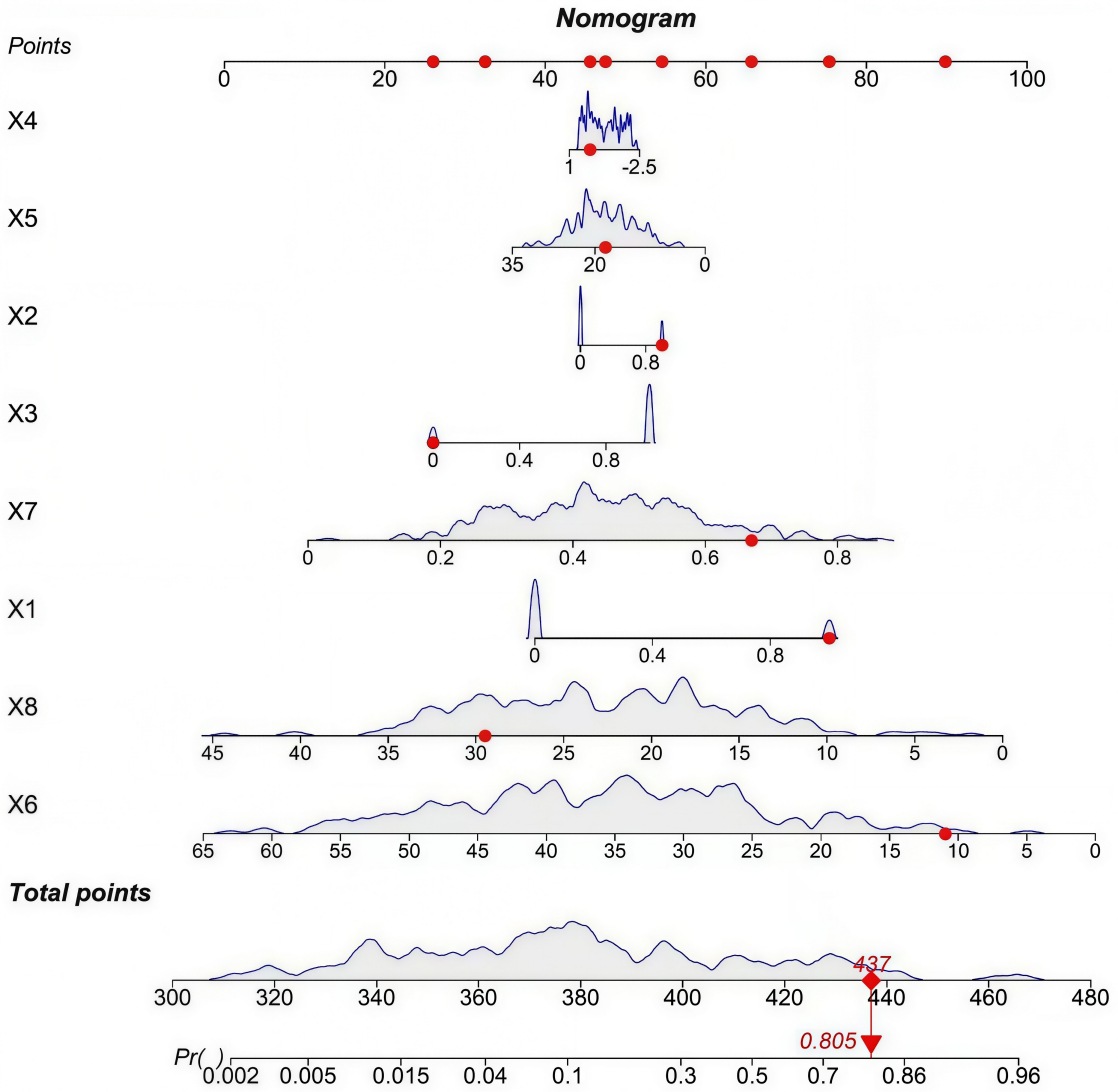
Nomogram for predicting osteoporosis treatment efficacy. X1, Comorbid Diabetes; X2, History of Fragility Fracture; X3, Glucocorticoid Use ≥ 6 months; X4, Femoral Neck BMD T-score; X5, Serum OC (ng/mL); X6, Serum PINP (ng/mL); X7, Serum β-CTX (ng/mL); X8, Serum 25(OH)D (ng/mL).

Furthermore, to facilitate rapid individualized risk assessment in clinical practice, a nomogram was constructed based on RF model (Figure 6). Clinicians can assign points for a patient’s specific values of each indicator on the corresponding axis. The sum of these points is then located on the Total Points axis, allowing for direct reading of the predicted probability of poor effect of treatment for that patient on the Risk Probability axis below.

## Discussion

This study developed and validated a machine learning model for predicting the risk of poor response to anti-osteoporosis therapy after 12 months in patients with primary osteoporosis by integrating baseline clinical characteristics, bone mineral density, and a panel of novel biomarkers reflecting the status of multiple bone metabolic pathways. Among the models, the RF model demonstrated the best overall performance, maintaining good discrimination (AUC = 0.825), calibration (Brier score = 0.148, Hosmer-Lemeshow test *P* = 0.385), and clinical utility in the independent validation set.

The core innovation of this study lies in the first systematic, multi-dimensional integration of markers reflecting bone formation, bone resorption, vitamin D status, and classic clinical risk factors to construct a predictive model for osteoporosis treatment efficacy. The eight independent predictors identified in this study elucidate key mechanisms influencing the response to anti-osteoporosis treatment from different dimensions. Serum β-CTX was confirmed by SHAP analysis as the strongest risk predictor. β-CTX is a specific marker of bone resorption, and its elevated level directly reflects increased osteoclast activity and high bone turnover rate (7). Our results showed that for every 1 ng/mL increase in serum β-CTX level, the risk of poor treatment response increased approximately 5.9-fold (OR = 5.879). This aligns with the fundamental theory of bone metabolism: under conditions of extremely active bone resorption, even potent anti-resorptive agents (e.g., bisphosphonates) may struggle to rapidly reverse the imbalanced bone microenvironment, leading to insufficient net bone gain (8). It should be noted that the threshold value of serum β-CTX (0.45 ng/mL) found in this study is a preliminary result based on a single-center retrospective cohort, and its clinical applicability needs to be verified by multi-center prospective studies with larger sample sizes and more diverse populations. Therefore, the clinical interpretation of this threshold effect should be kept cautious, and it cannot be directly used as a clinical diagnostic or treatment indicator at present. Conversely, serum PINP, reflecting bone formation activity, exhibited a protective effect, with higher levels associated with a lower risk of poor response (OR = 0.906). This indicates that patients with a baseline reserve of osteogenic function might respond better to bone-forming agents (e.g., teriparatide) or treatment regimens with anabolic effects (9). Regarding systemic regulatory factors, insufficient serum 25(OH)D was another significant independent risk factor (OR = 0.861). Vitamin D deficiency not only impairs intestinal calcium absorption, leading to insufficient mineralization substrate, but its active form also directly regulates osteoblast differentiation and function (10). In this study, the mean 25(OH)D level in the poor-response group was only 18.25 ng/mL, which is definitively insufficient. This finding strongly suggests that actively correcting vitamin D deficiency to a sufficient level (typically recommended > 30 ng/mL) before initiating any anti-osteoporosis pharmacotherapy may be a fundamental and necessary step to optimize overall treatment efficacy (11, 12).

Among clinical characteristics, comorbid diabetes increased the risk of poor response by 2.8-fold (OR = 3.784). The skeleton in diabetic patients is often in a “glucotoxic” environment, where the accumulation of Advanced Glycation End-products (AGEs) impairs collagen cross-linking, chronic low-grade inflammation, and impaired Insulin-like Growth Factor-1 (IGF-1) signaling collectively contribute to reduced bone quality and impaired repair capacity (13, 14). Therefore, for osteoporotic patients with diabetes, stricter glycemic control and consideration of more potent or combination therapies may be required (15, 16). A history of fragility fracture, a marker of skeletal fragility and severely compromised bone microarchitecture, was confirmed as a risk factor (OR = 1.226) in our model, emphasizing that these patients belong to a very high-risk group requiring more aggressive and individualized treatment (17).

Notably, a series of novel biomarkers detected in this study, such as DKK1, sclerostin, OPG, and RANKL, although involved in the key Wnt signaling pathway and the OPG/RANKL/RANK system, respectively, did not demonstrate independent predictive value beyond traditional markers (β-CTX, PINP) and basic nutritional status (25(OH)D) in the model predicting 12-month BMD response (18). This may indicate that for predicting relatively short-term treatment response (BMD changes), “effector markers” directly reflecting the rates of bone resorption and formation are more predictive than upstream “regulatory factors” (19, 20). One possible explanation is that changes in upstream regulators tend to be slower and are subject to complex feedback mechanisms, making their serum levels less correlated with short-term fluctuations in bone turnover. In contrast, effector markers directly reflect the current activity of osteoclasts and osteoblasts, capturing early changes following therapeutic intervention more sensitively. Moreover, the timing of measurement (baseline only) might not be optimal for these novel biomarkers to exert their predictive potential; dynamic monitoring of their trajectories could provide additional insights. Nevertheless, this does not preclude the potential value of these novel biomarkers in other contexts, such as predicting long-term fracture risk, assessing the efficacy of specific targeted therapies (e.g., anti-sclerostin antibodies), or reflecting bone quality, which warrants further investigation in future studies (20, 21)

Methodologically, this study has several strengths. First, in order to enhance the simplicity and robustness of the model, the progressive variable selection strategy of “univariate analysis, LASSO regression and multivariate logistic regression” was used in this study. Firstly, the potential related variables were preliminarily screened by univariate analysis. Furthermore, LASSO regression was introduced to effectively avoid the risk of overfitting and eliminate multicollinearity between variables by using its coefficient shrinkage property. Although LASSO regression did not further reduce the number of variables in this study, it verified the independent predictive value of each of the 8 variables screened by univariate analysis, which laid a reliable foundation for subsequent multivariate logistic regression analysis and reflected the key advantages of this method compared with single factor screening (22). Second, we constructed and compared three different machine learning models, finding that the RF model had the strongest capability to capture non-linear relationships and interactions within complex data, a performance confirmed in the validation set. Most importantly, the introduction of SHAP value analysis successfully addressed the common “black box” dilemma of machine learning models.

The RF model is the optimal prediction model in this study with the best discriminatory ability and calibration, while the nomogram is a clinical transformation tool based on the core 8 independent predictive factors of the RF model. Although the nomogram is inherently regression-based, its core variables are consistent with the RF model, which ensures that the nomogram can retain the main predictive value of the RF model while realizing the rapid and simple clinical application, and this is the key conceptual link between the two tools. However, the clinical applicability of the model and nomogram still has certain limitations. Some predictors in the model, such as serum β-CTX, PINP, and 25(OH)D, need specialized detection technologies such as electrochemiluminescence and high-performance liquid chromatography, which are not routinely available in many primary clinical settings, thus limiting the real-world implementation of the model in grassroots medical institutions. In order to solve this problem, we will carry out follow-up research to screen the key predictors that can be detected by routine clinical assays and simplify the prediction model on the premise of ensuring predictive performance; at the same time, we will cooperate with primary medical institutions to popularize the detection technology of core bone metabolism biomarkers, so as to improve the clinical applicability and promotion value of the model in different levels of medical institutions.

The definition of poor treatment response in this study integrated limited BMD gain, incident fracture and treatment regimen adjustment, which may have heterogeneity due to the mixture of biological non-response and clinical decision-making/tolerability issues. Although we adopted a double-blinded independent evaluation method with senior expert arbitration to minimize subjective bias, this definition still has potential limitations. In future studies, we will further optimize the outcome definition by separating biological non-response from clinical factors and increasing the follow-up time to reduce the impact of stochastic events such as short-term fractures. In addition, the 3% BMD increase threshold adopted in this study is based on mainstream clinical guidelines, but its clinical relevance across different skeletal sites (e.g., lumbar spine vs. femoral neck) and treatment modalities (e.g., bisphosphonates vs. parathyroid hormone analogs) is still debatable. In future research, we will further explore the optimal BMD threshold for different skeletal sites and treatment modalities to improve the accuracy of efficacy evaluation. The treatment regimen adjustment in the definition of poor treatment response is partially subjective and physician-dependent, which is a potential confounding factor for outcome classification. In this study, 30.00% of the treatment adjustments were due to drug intolerance, and 70.00% were due to poor efficacy. Although the proportion of intolerance-induced adjustment is relatively low, it still may have a slight impact on the accuracy of outcome classification. In future studies, we will exclude the cases of treatment adjustment only due to drug intolerance from the ineffective treatment group to further reduce the confounding effect of subjective factors.

However, this study also has several limitations. First, the limitation of this study is the complete lack of external validation in independent cohorts, which makes the generalizability and robustness of the research results uncertain. In addition, as a single-center retrospective study, the patient source is relatively homogeneous, potentially introducing selection bias; the current validation strategy only adopts the 7:3 random split within the same center, which cannot fully address optimism bias and institutional effects, and further affects the objective evaluation of the model’s actual performance. Therefore, the core research plan of our next step is to carry out multi-center prospective research, and fully verify the external validity and robustness of the model in independent cohorts from different regions, different populations, different medical institutions and even different countries. Second, this study only included baseline pre-treatment data as predictive factors, and did not consider important dynamic factors such as patient treatment adherence, early biomarker changes within 3–6 months of treatment, and drug dose adjustments during follow-up, which are known to substantially influence the treatment response of osteoporosis. Due to the retrospective nature of the study, it is difficult to obtain complete and standardized dynamic factor data from the hospital electronic medical record system, which limits the comprehensiveness of the prediction model. In future prospective studies, we will design a standardized data collection protocol to collect dynamic factor data, and integrate temporal data and mobile health technologies to build a dynamic prediction model, so as to further improve the model’s predictive accuracy and clinical applicability. Third, the model incorporated only baseline pre-treatment data and did not account for dynamic factors during treatment that might influence efficacy, such as biomarker fluctuations, patient adherence, and lifestyle interventions. Future research could explore integrating temporal data and mobile health technologies to build dynamic prediction models.

In conclusion, the integrated prediction model developed and validated in this study (with the RF model being optimal) can accurately identify patients at high risk for poor response to anti-osteoporosis therapy before treatment initiation. This model combines good predictive performance with clinical interpretability.

## Data Availability

The original contributions presented in the study are included in the article/Supplementary material, further inquiries can be directed to the corresponding author.
