# Supplementary material for: Predicting poor response to anti-osteoporosis therapy: a machine learning model integrating clinical and novel biomarker data
Source: Front Med (Lausanne). 2026 May 14;13:1786209. doi: 10.3389/fmed.2026.1786209 (PMC13216482; doi:10.3389/fmed.2026.1786209)
Supplement: Supplementary file 1 [file Table_1.docx]

**Basic principles and application characteristics of three machine learning methods**

Three classic machine learning algorithms were selected to construct the prediction model for osteoporosis treatment response, and their basic principles and application characteristics are as follows: (1) Random Forest (RF): an integrated learning algorithm based on multiple decision trees, which uses bootstrap sampling to construct multiple decision trees and takes the majority voting result as the final prediction. It has strong anti-overfitting ability and can effectively capture the nonlinear relationship and interaction between multiple variables, which is suitable for the analysis of clinical high-dimensional data with multiple confounding factors. (2) Support Vector Machine (SVM) with radial basis function kernel: a supervised learning algorithm that maps the original data to a high-dimensional feature space through the kernel function to solve the linear inseparable problem. The radial basis function kernel is the most commonly used kernel function, which has good adaptability to non-linear clinical data and strong generalization ability for small sample data. (3) K-Nearest Neighbors (KNN): a lazy learning algorithm that does not need to train the model in advance, and predicts the category of the test sample according to the category of the K nearest training samples. It is simple and easy to implement, and can reflect the local distribution characteristics of the data, which is suitable for the comparison of the prediction effect of the integrated learning algorithm and the traditional machine learning algorithm. The above three methods were selected because they can cover different types of machine learning algorithms (integrated learning, kernel method, lazy learning) and are widely used in the construction of medical clinical prediction models, which is convenient for the comprehensive comparison of the prediction performance of different algorithms.
